# Embedded EPICS server for PowerPMAC motion controllers

**DOI:** 10.1088/1748-0221/21/01/p01002

**Published:** 2026-01-05

**Authors:** Oleg Makarov, Sergey Stepanov

**Affiliations:** a Advanced Photon Source, Argonne National Laboratory, 9700 S. Cass Ave, Bldg.436D, Argonne, Illinois, 60439, U.S.A.

**Keywords:** Beam-line instrumentation (beam position and profile monitors, beam-intensity monitors, bunch length monitors), Hardware and accelerator control systems, Instrumentation for neutron sources, Instrumentation for synchrotron radiation accelerators

## Abstract

An embedded server layer of Experimental Physics and Industrial Control System (EPICS) for PowerPMAC motion controllers has been developed and deployed at two undulator beamlines of the National Institute of General Medical Sciences and the National Cancer Institute (GM/CA) Structural Biology Facility at the Advanced Photon Source (APS). This compact, open source solution makes the power and versatility of PowerPMAC motion controls directly accessible to distributed EPICS clients. At GM/CA the system controls about 200 servo and stepper motors — both encoded and unencoded — and multiple digital and analog I/O accessories. The server stack comprises two sublayers: a lower-level driver and database that communicates directly with PowerPMAC, and a facility-specific soft sublayer built on top. The paper describes installing EPICS on PowerPMAC, the implementation of both layers and client examples, including on-the-fly scanning.

## 1 Introduction

Motion control is a core component of beamline control and data acquisition at synchrotron radiation facilities. Beamlines commonly deploy around 100 motors of various types — stepper, brushed and brushless servo, piezo, etc. — with options for no encoder, incremental encoders, or absolute encoders. Beamline operations often require synchronized motions of multiple motors to execute trajectories or perform on-the-fly scans coordinated with external devices such as detectors or shutters. These needs call for a flexible, highly configurable motion controller. The PMAC family of motion controllers became popular due to their distributed, modular design and programmable configuration.

At the APS, we have operated several PMAC generations integrated with EPICS-based distributed beamline controls [1, 2]. Our first systems used 16 axis PMAC1-VME controllers with an EPICS driver by Tom Coleman [3]. We then upgraded to 32 axis Turbo PMAC2-VME Ultralite controllers and developed an open source EPICS driver [4] later adopted at several APS Sectors and at Diamond Light Source. By 2023, as the APS accelerator and beamlines were preparing for a major upgrade, it became clear that the VME electronics format had become outdated. Our team, GM/CA at the APS [5], selected the 128-axis PowerPMAC as the modern replacement.

PowerPMAC is a compact, standalone networked controller. In our implementation we use Power UMAC with an ARM CPU, 2 GB of RAM, internal flash storage, and a USB 3.0 slot for external storage [6]. It runs embedded Debian Linux 8 (Jessie) and hosts a daemon (powerpmac) that interfaces to the motion control functionality. Because the PowerPMAC control commands and motor tuning parameters differ substantially from earlier PMAC generations, we needed to integrate the controller with EPICS. Specifically, we developed an EPICS Input/Output Controller (IOC) residing in PowerPMAC and translating EPICS database operations into PowerPMAC commands and vice versa, thus enabling EPICS clients to access motion controls over the local network.

We were aware of EPICS IOC implementations that run on a separate computer and communicate with PowerPMAC over SSH [7, 8]. However, our tests of the EPICS ai record read/write latencies showed 40μs for local access versus 2500μs over the network — a factor of 62 slower. One could argue that EPICS clients would access EPICS records over the network anyway, but in that case there would be two network transactions instead of one. In addition, clients typically access only a few EPICS records at a time as not all 128 motors are likely to be moved simultaneously, whereas EPICS IOC must continuously synchronize *all* records with PowerPMAC. For example, the IOC needs to poll positions and status flags of all motors at 10 Hz. We also considered the overhead of SSH encryption/decryption, which could further degrade performance. Finally, we preferred a compact solution without an additional computer.

EPICS is widely used at synchrotrons including APS, the Australian Synchrotron, the Canadian Light Source, NSLS-II, Diamond Light Source, DESY, the Swiss Light Source, SSRF, and others. PMAC controllers, in turn, offer powerful, configurable motion control that unifies heterogeneous motor and encoder types and supports multi-axis synchronization. Given PMAC’s popularity at beamlines, we expect our open-source development to be broadly useful.

## 2 Installing EPICS base in PowerPMAC motion controller

Before implementing and deploying the PowerPMAC EPICS driver, we installed several additions to Debian Jessie on the controller. At a minimum, we added the NTP daemon to synchronize the PowerPMAC clock with EPICS clients and the screen package to run the EPICS IOC without a connected terminal. For smoother compilation of EPICS, we also updated cpp, gcc, libc6-dev, libgcc-dev, libstdc++6, re2c, unzip, and zlib. All of these packages are in the Debian Jessie repository; no third-party software is required. Because Jessie is from 2015, apt sources must point to http://archive.debian.org.

Compiling EPICS requires about 300 MB of storage. In principle, EPICS can be built on the internal flash. However, flash media have limited write/erase cycles, and the PowerPMAC internal storage is mounted read-only by default for longevity. To reduce wear, simplify backups, and provide room for future additions, we installed EPICS on an external 128 GB USB 3.0 flash drive.

EPICS is open source and distributed as EPICS Base [9] and synApps (synchrotron applications) [10]. EPICS Base provides the build environment and libraries for servers and clients; for servers, it also includes the database manager and Channel Access server. The database manager maps hardware parameters to EPICS database fields, and Channel Access server provides remote read/write access to the database for clients. synApps is a collection of EPICS modules that help the database manager interface to hardware such as motion controllers, scalers, and I/O electronics. When building an EPICS IOC, one includes and compiles only the synApps modules needed for the target hardware.

EPICS Base supports a wide variety of computer CPUs. On PowerPMAC, the CPU was auto-detected as linux-arm, and the latest version 7.0.9 of EPICS Base compiled cleanly without special configuration. The compilation was fast and no cross-compiling was necessary. Before compiling synApps, we defined standard EPICS environment variables, such as EPICS_BASE (the EPICS Base install path) and EPICS_HOST_ARCH (linux-arm), and added them to /etc/bash.bashrc on PowerPMAC, as described in [11].

In addition to the *ppmac* driver and *psub* add-on (described below), we included the following standard EPICS synApps modules in the PowerPMAC IOC:

1. asyn [12] for communicating with the PowerPMAC controller
2. busy [13] for motor scanning support with EPICS scan record
3. calc [14] for axes transformations support
4. autosave [15] for saving/restoring motors parameters

These modules compiled without issue on PowerPMAC, which runs mainstream Debian Linux. Older PowerPMACs contain a PowerPC CPU auto-detected by EPICS as linux-ppc. The system works there too with the exception that it needs EPICS Base version 7.06 or older because it operates under older Debian Linux 5 (Lenny).

## 3 Embedded EPICS driver and databases for PowerPMAC

The EPICS driver and databases for PowerPMAC are built using the AsynPortDriver framework [4, 16] and the PowerPMAC API. AsynPortDriver provides a convenient interface between driver code and EPICS database variables. The PowerPMAC API is documented via two header files on the controller: /opt/ppmac/libppmac/gplib.h and /opt/ppmac/rtpmac/RtGpShm.h. These self-documenting files define C-language functions and shared-memory pointers for accessing motion controller parameters. The corresponding functions reside in the shared library /opt/ppmac/libppmac/libppmac.so, which must be linked to the EPICS IOC application. The Makefile and source code for our *ppmac* driver are open source and available from the GM/CA website [11].

In the PMAC family of controllers, including PowerPMAC, the drives are arranged into coordinate systems where they are driven by PMAC motion programs. All motors in a coordinate system share common start/abort commands, and the controller synchronizes their motion at the internal clock rate (2 kHz for PowerPMAC). As a result, EPICS motor record [17], which targets individual motors, is not an ideal fit. Instead, starting with PMAC1-VME in 1996 we have used generic EPICS ai, ao, bi, bo, mbbo, lso, longin, and waveform records to access various PMAC parameters and this approach worked well. We, therefore, adopted the same strategy for PowerPMAC. The EPICS database of the *ppmac* module consists of controller records (MC.db), coordinate system records (CS.db), and drive records (CSA.db and MTR.db), see [11]. The controller database contains only 4 records mapping PMAC servo timer status, command string, response string, and error string. For each coordinate system, CS.db contains 16 records mapping the start and abort commands, two coordinate system status words, motion program selection, motion program state, autostart switch, acceleration times, command and response strings, and few others. Finally, for each motor we load 6 records in CSA.db and 18 records in MTR.db. These include requested and actual motor positions, jog, program, and homing speed settings, actual velocity, scale and offset factors for converting encoder data to physical units, motor following error value, two motor status words, and more. Typical *ppmac* module records look as follows:


record(ai, “$(CS)$(A)ActPos”) {
   field(DTYP, “asynFloat64”)
   field(INP,  “@asyn($(PORT),$(N),$(TIMEOUT))C$(PA)_POS”)
   field(PREC, “4”)
   field(SCAN, “I/O Intr”)
}
record(ao, “$(CS)$(A)RqsPos”) {
   field(PINI, “1”)
   field(DTYP, “asynFloat64”)
   field(OUT,  “@asyn($(PORT),$(N),$(TIMEOUT))C$(PA)_RQS”) 
   field(PREC, “4”)
   info(asyn:READBACK,”1”)
}


Here ActPos and RqsPos are the records corresponding to the actual and requested motor positions, respectively. $(CS) is the EPICS database name of the coordinate system; and $(A) is the EPICS database name of the PowerPMAC drive. $(PA) is the axis name in the PowerPMAC coordinate system. Although PowerPMAC coordinate system can support up to 32 axes, the current EPICS driver is limited to four axes (X,Y,Z, and A), which has been sufficient for our applications, but can be easily extended. $(PORT) is the name of asyn port (the same for all records), and $(N) is the coordinate system number in PowerPMAC. The strings C$(PA)_POS and C$(PA)_RQS are unique labels in the PowerPMAC driver (see ppmacDrv.h [11]) that the driver uses to map the corresponding asyn records to the OMRON powerpmac daemon running in the controller.

With the above databases one can drive motors grouped into PowerPMAC coordinate systems or jog individual motors. For each assembly of 1,2 or 3 drives the system includes a choice of several motion programs with different options for handling backlash. These motion programs are loaded into and saved in the PowerPMAC system. The simplest motion program for a two-drive coordinate system looks as follows:


open prog 30
//=============================================================
// Program-30: two motors, slew (no backlash)
// AccelTime -> TA -> Coord[L0].Ta
// AccelSCurve -> TS -> Coord[L0].Ts
// X_RqsPos -> X -> Q1024
// Y_RqsPos -> Y -> Q1028 
//-------------------------------------------------------------
  L0=Q70                 //coordinate system number
  TM(-1)                 //feedrate mode, 1.0 axis unit / time unit 
  TA(Coord[L0].Ta)       //set acceleration time 
  TS(Coord[L0].Ts)       //set s-curve part of acceleration time 
  FRAX(X,Y)              //specify feedrate axes
  LINEAR                 //linear-interpolation move mode
  ABS                    //absolute move mode
  X(Q1024) Y(Q1028)      //goto XY RqsPos
  DWELL0                 //wait until move is completed
close


Here the PowerPMAC Q-variables are loaded from EPICS records with the help of the *ppmac* driver.

**Figure 1.**
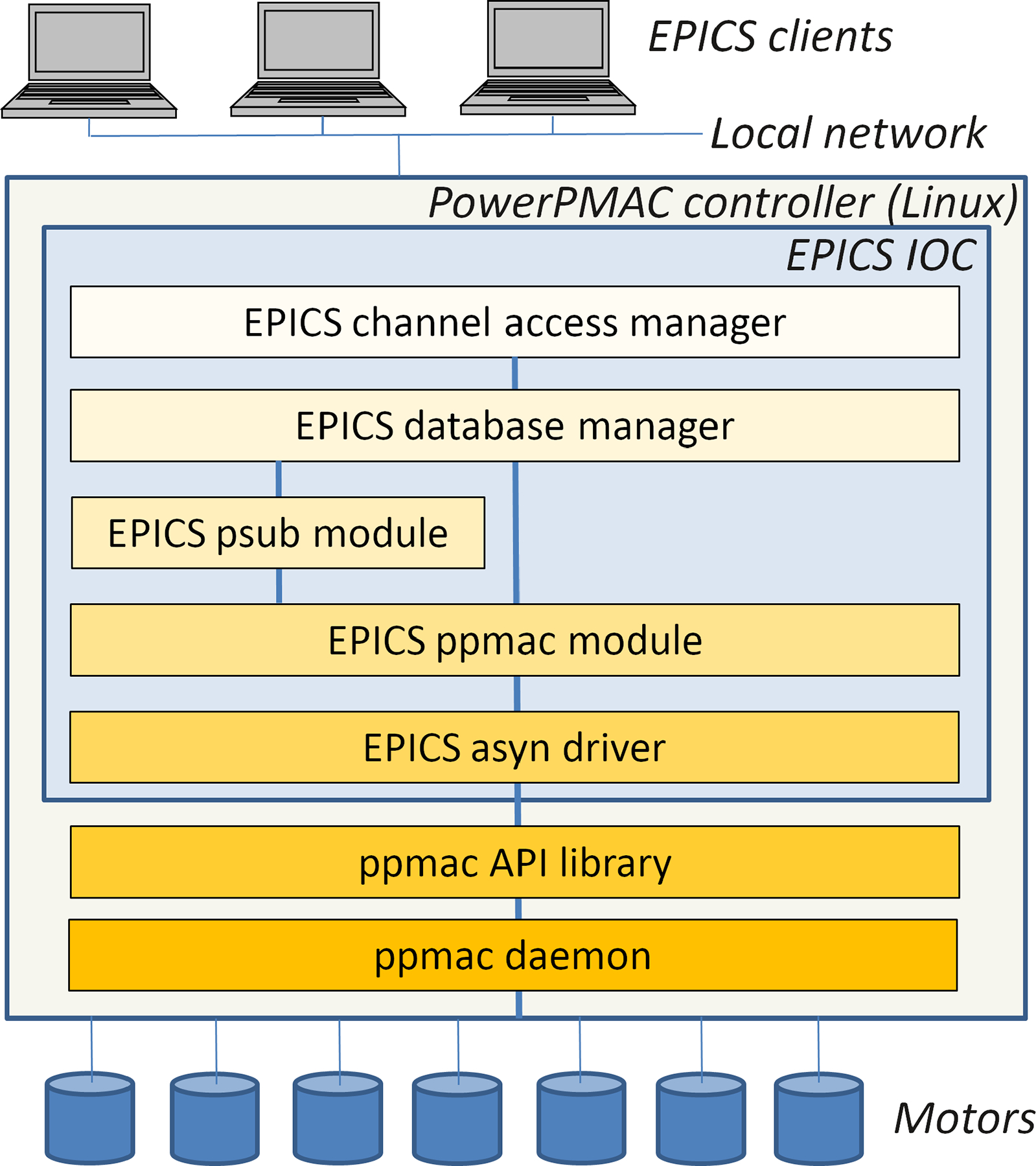
The structure of embedded EPICS support for PowerPMAC motion controllers.

Figure 1 illustrates the EPICS layer hierarchy in our application. The optional *psub* module is described in the next section. The powerpmac daemon at the bottom of figure 1 is part of the proprietary control software supplied with PowerPMAC controllers.

PowerPMAC controllers are supplied with Integrated Development Environment (IDE) software [18] that can be installed on a Microsoft Windows computer and used to control PowerPMAC over SSH. We use this graphical tool for initial setup and motor tuning; it provides tools for optimizing parameters (e.g., minimizing following error). The IDE is not required and is not used in routine operations.

**Figure 2.**
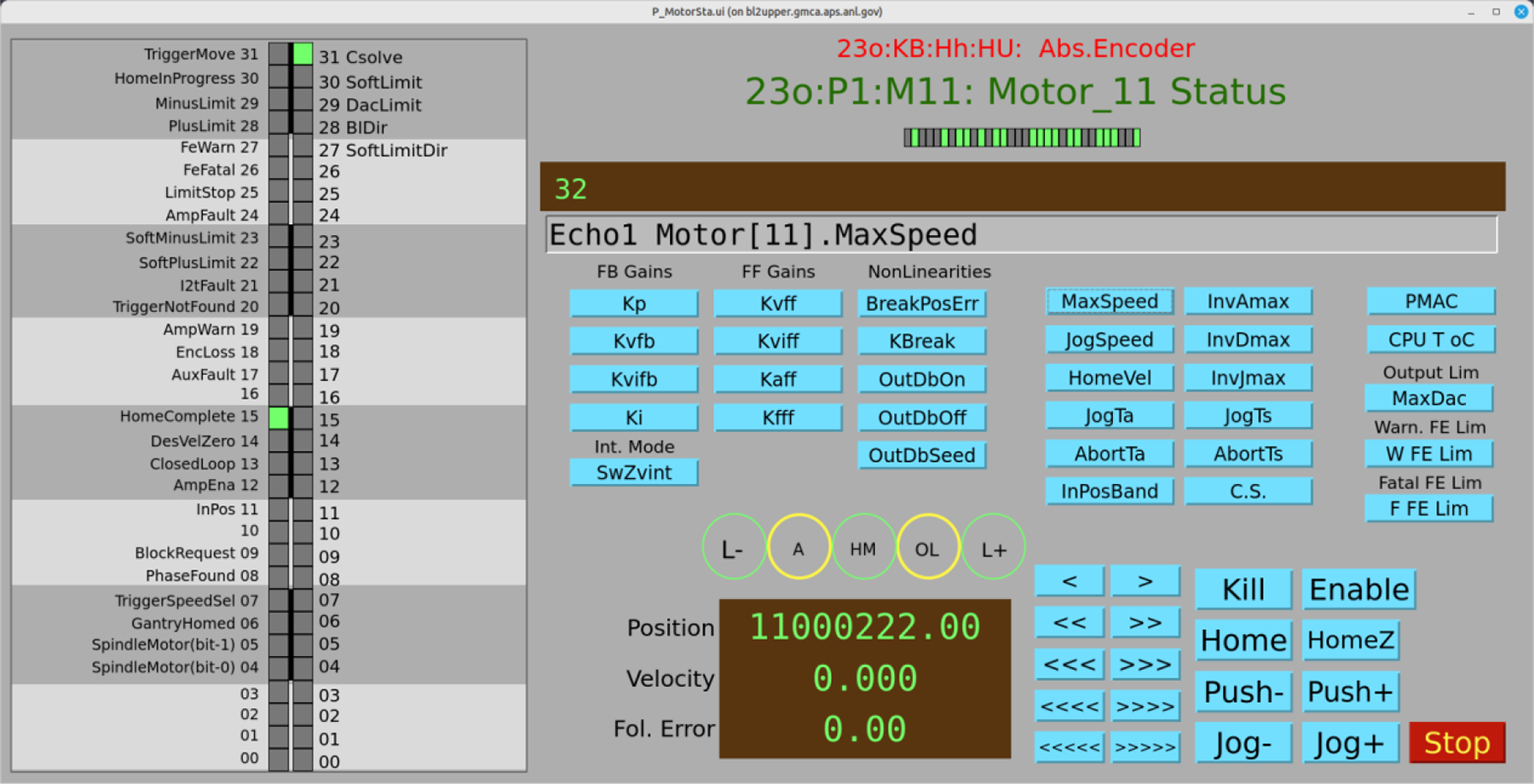
Example EPICS status and control screen for a PowerPMAC motor. These screens can be launched on any EPICS client on the local network using MEDM or CaQtDM EPICS display programs.

**Figure 3.**
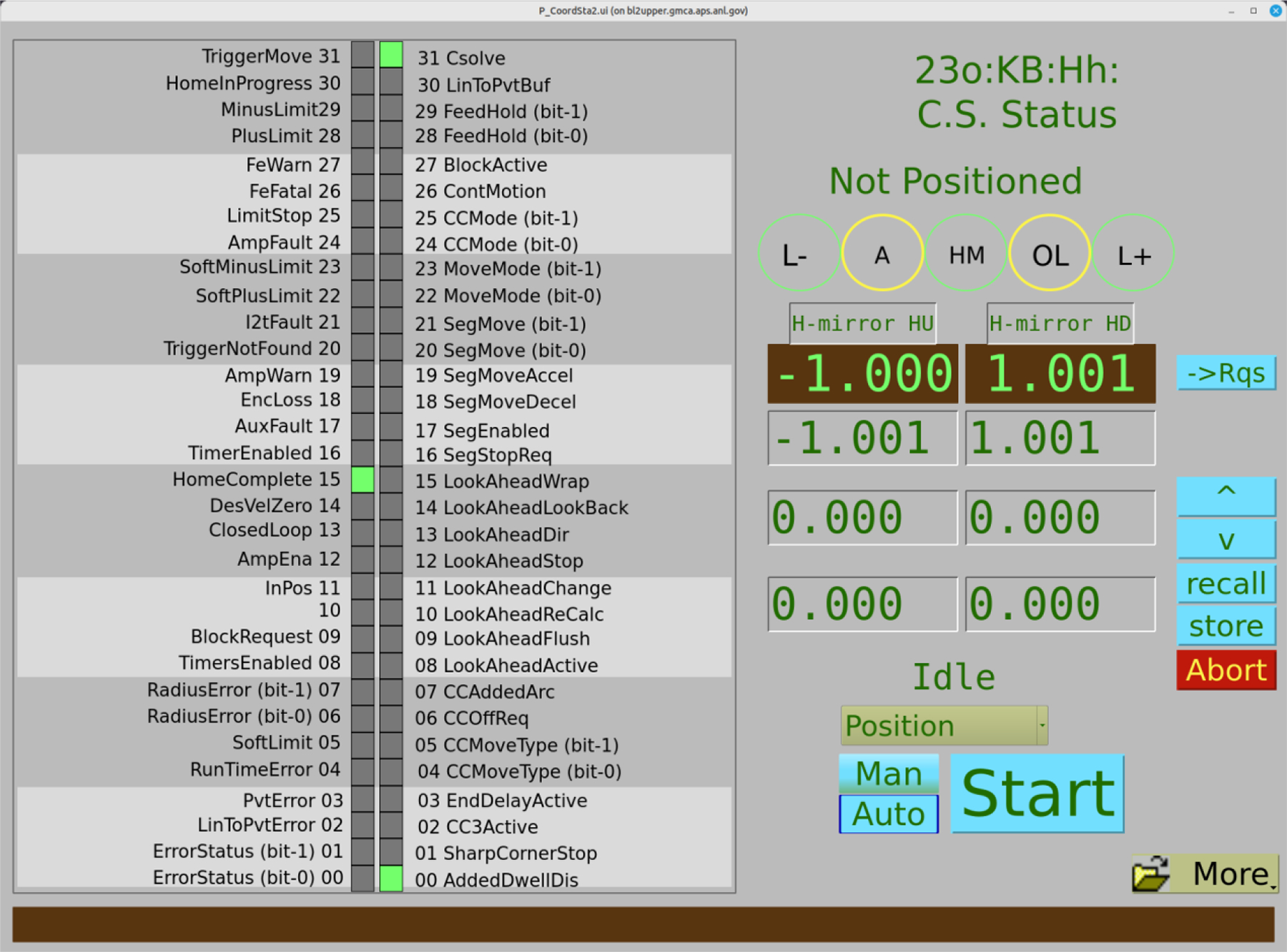
Example EPICS status and control screen for PowerPMAC coordinate system assembly consisting of two motors.

The *ppmac* module exposes EPICS access to PowerPMAC controller parameters, individual drive motion parameters (figure 2), and coordinate systems — groups of motors defined to run synchronously (figure 3). It also provides controls for digital and analog I/O cards, which are optional PMAC accessories. All parameters are mapped to EPICS database fields commonly called “process variables” (PVs). For example, *ppmac* provides PVs for actual/requested motor positions, status words for motors and assemblies, start/stop commands, and more. Thus, *ppmac* provides a generic foundation for EPICS-based control of PowerPMAC.

## 4 Facility software layer on top of *ppmac* and client examples

**Figure 4.**
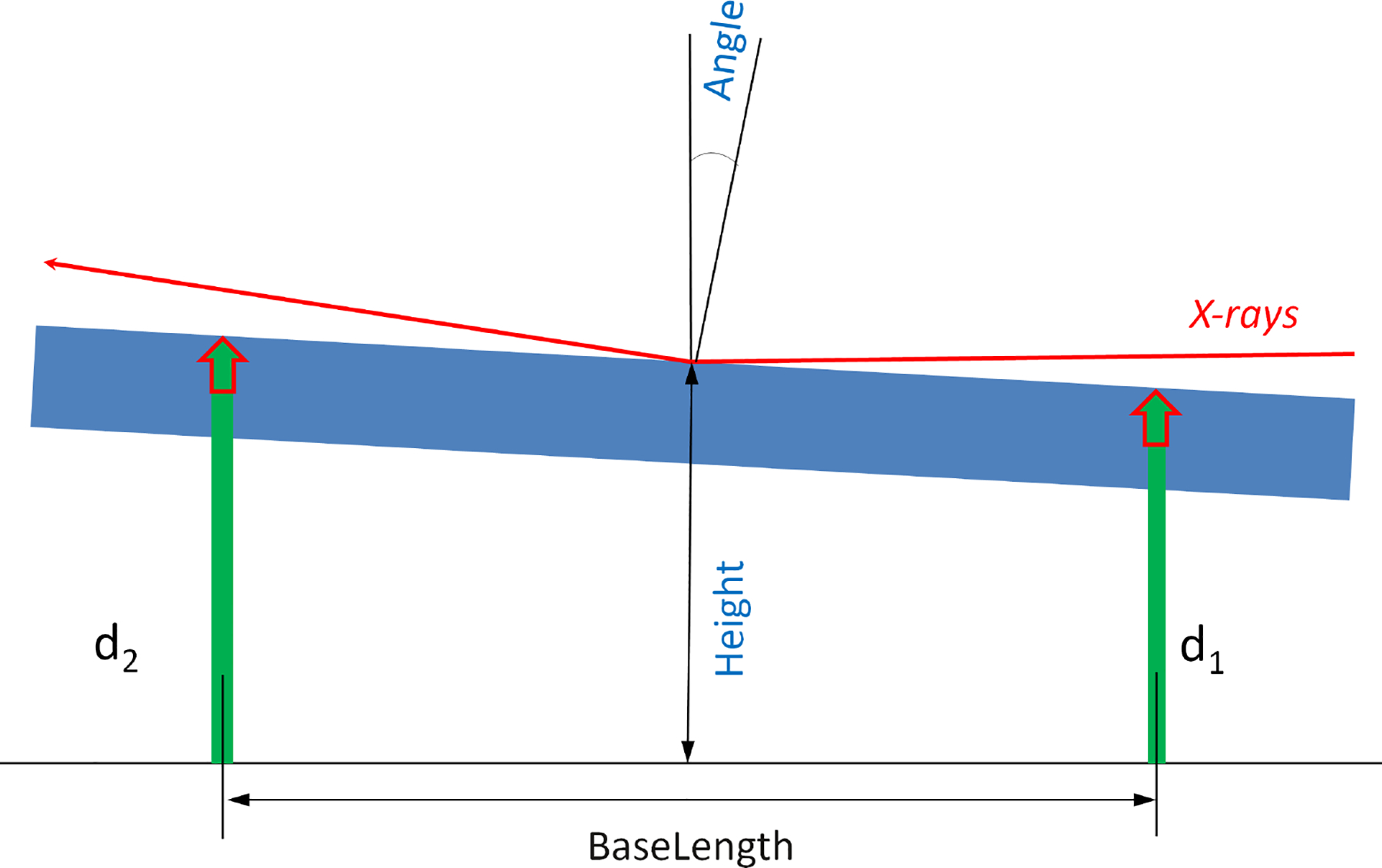
Mirror support assembly. PMAC provides synchronous motion of drives *d*_1_ and *d*_2_ while end user is interested if driving mirror height and angle.

With *ppmac* one can jog individual motors (figure 2) or synchronously drive several motors arranged into coordinate system (figure 3). However, in many cases there may be requirements to drive combined axes. In the synchrotron beamline operations a simple example is two-jack mirror support where one needs to drive mirror height and angle (figure 4). Other examples include:

- Three-jack table support providing height, pitch, and yaw
- Slits with individual blades defining center and width
- Three-motor support for positioning area detector
- Double-crystal monochromator (DCM), where the second crystal’s motion is slaved to the Bragg angle of the first crystal and users dial X-ray energy or wavelength rather than angle

In theory, these composite axes could be implemented within the PMAC via custom motion programs. While that may be necessary for applications with critical trajectories, we find it more convenient for typical synchrotron beamline operations to implement these transformations at the EPICS layer. EPICS-level transformations are easier to track, debug, and modify, since all parameters are exposed via EPICS display screens.

The *psub* module constitutes the second part of our EPICS interface to PowerPMAC and supplements *ppmac* by providing support for composite axes:

1. Recalculation of actual and requested positions of individual drives into corresponding composite-axis positions
2. Recalculation of requested composite-axis positions into requested positions of individual drives
3. Propagation of limit switches and amplifier status flags from individual drives to composite axes
4. Recalculation of motion speeds between axes and drives

These calculations are performed in a *tsub* EPICS record [4, 11], a transformation record with multiple inputs and outputs. For each type of combined-axes assembly (mirror, DCM, table, slit), the *tsub* record is supplied with a small, assembly-specific transformation routine (typically 20–30 lines of *C* code) [11]. Separate EPICS records handle each axis-related field (actual and requested positions, limit switch flags, speeds). None of the *psub* layer records — including *tsub* — communicate directly with the PowerPMAC API; they interact only with *ppmac* EPICS records.

**Figure 5.**
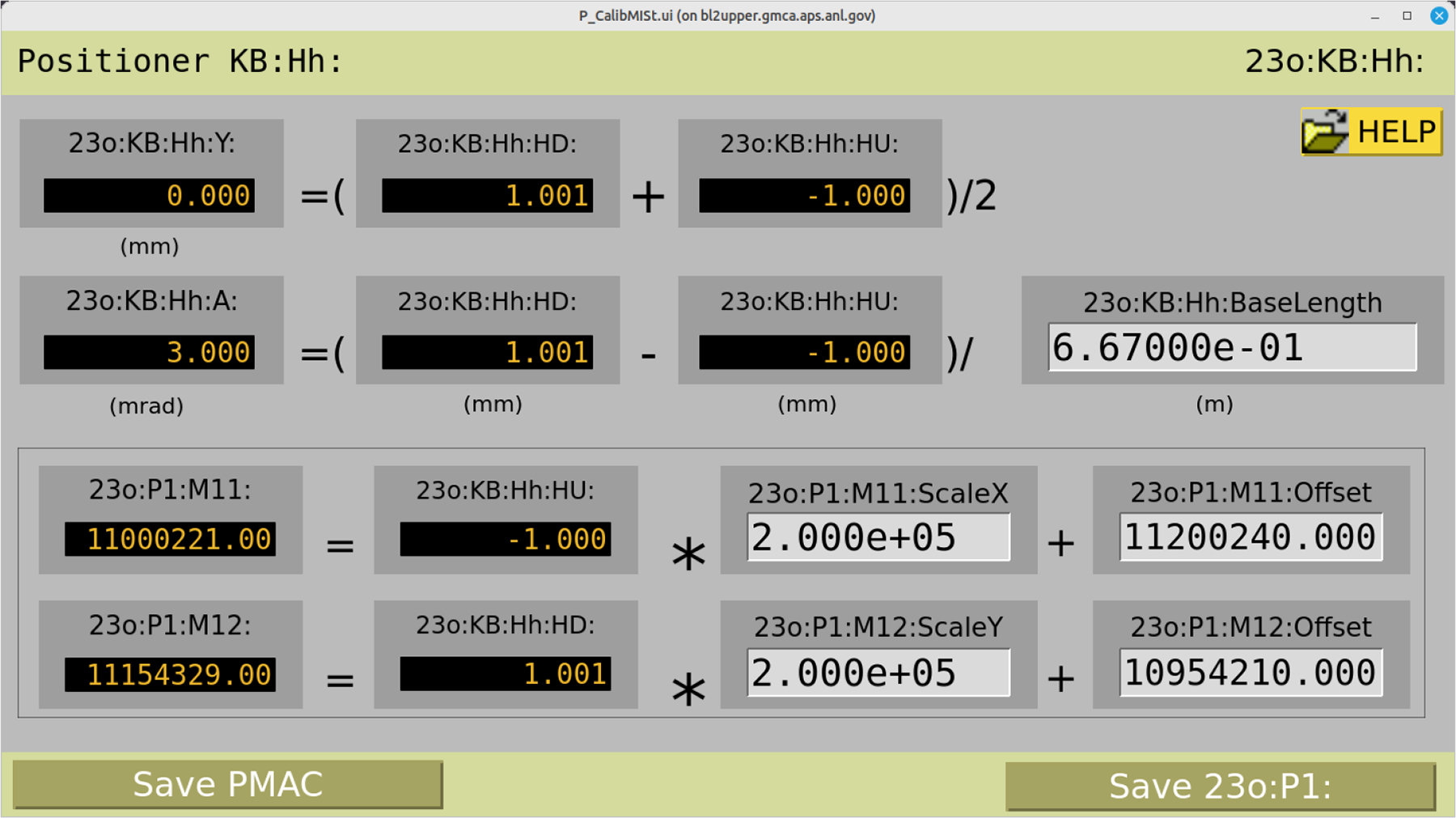
EPICS calibration screen for a mirror assembly.

Figure 5 shows a calibration screen for a mirror assembly, where positions of the two jacks (HU and HD) are transformed into mirror height (Y) and angle (A). Baselength is a distance between the jacks; it serves as a calibration parameter. Because X-ray mirror angles are small, these transformations are linear, although exact trigonometric functions can be used when needed — for example, in converting Bragg angle to X-ray energy for a DCM.

**Figure 6.**
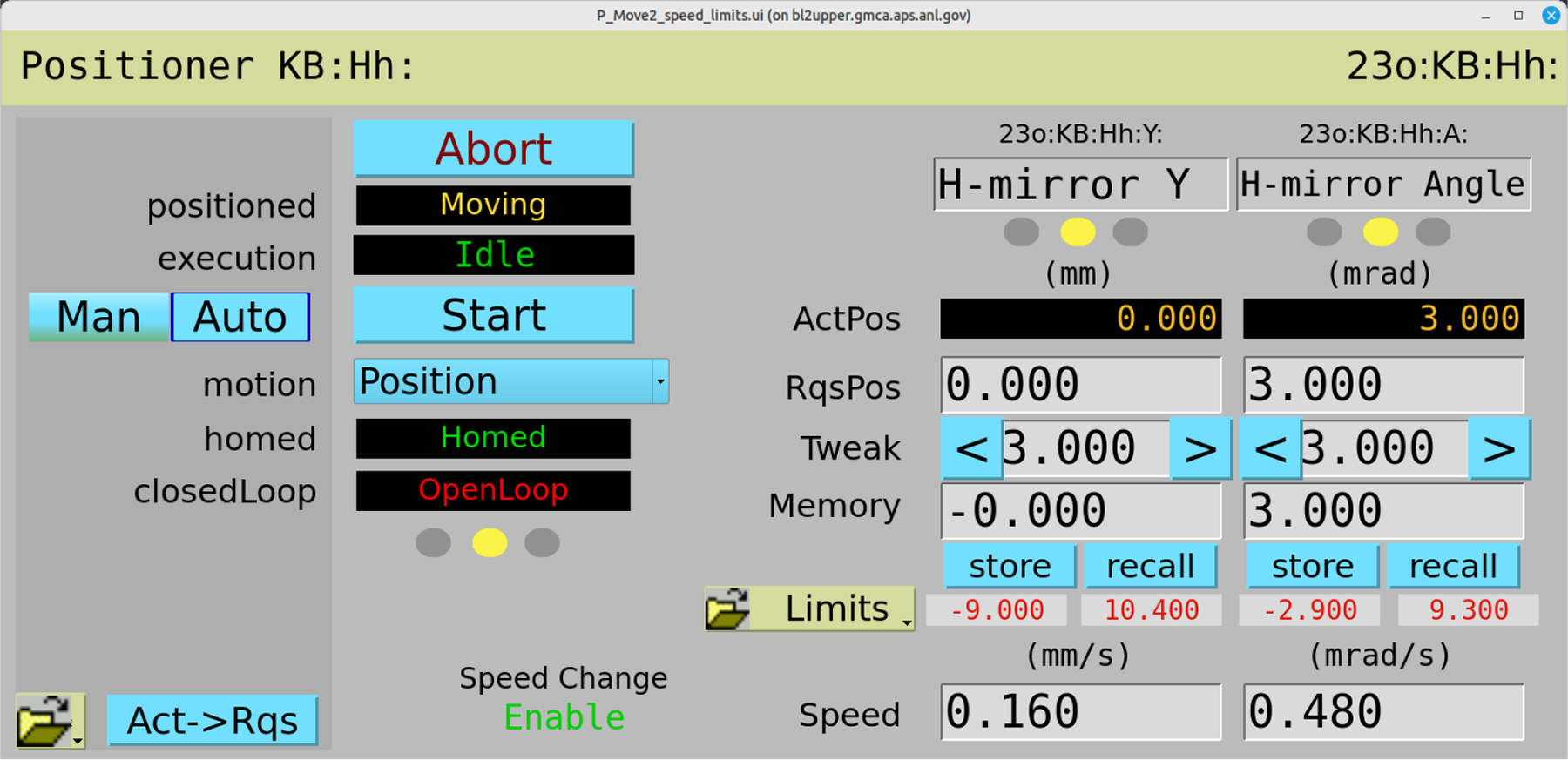
EPICS motion controls for a mirror assembly. The three dots are the indicators for lower-end limit, amplifier status and upper-end limit (from left to right).

From the end-user standpoint, operating PowerPMAC motor assemblies is straightforward (figure 6). Users dial requested positions (RqsPos). In Auto mode, motion starts immediately when any RqsPos changes. In Man mode, the user presses Start to initiate motion; this allows simultaneous changes to height and angle. Motion speed is easily adjustable, which is essential for fly scanning. Multiple motion programs are available via a drop-down menu. In our case, all are linear but differ in backlash handling. The remaining controls display status flags reported by PowerPMAC. The three indicator dots denote, from left to right, the lower limit, amplifier status, and upper limit.

## 5 Conclusions

We presented a compact, open-source solution for controlling PowerPMAC motion controllers via EPICS, available for free download from the GM/CA website [11]. The system has been successfully deployed at two beamlines in Sector 23 at the APS, with four PowerPMAC units controlling all motors. Peak CPU usage observed was under 12% of a single core (PowerPMAC has four cores) on a unit controlling 40 motors in 19 coordinate systems plus one analog I/O and three digital I/O PMAC accessories. The overall EPICS database for that unit included 2660 records. As discussed in section 3, the *ppmac* database per drive contains 40, 32, or 29 records for 1-, 2-, and 3-motor coordinate systems respectively. The *psub* module adds some more records for combined axes and the transformations, resulting in an average of 60–65 total records per physical drive.

The system has been used routinely for on-the-fly scans [19–22]. We have implemented both fixed-time and fixed-step modes. The fixed-step implementation, originally developed for a previous PMAC generation [16], links a virtual motor (a PMAC channel not driving real hardware) to the pulse stream sent to the scanned motor(s). The PMAC output for the virtual motor is routed to a small electronic module that converts step/direction signals to step-up/step-down signals. The latter serve as inputs to a multichannel scaler that synchronizes motion with detector output. In fixed-step mode, the PMAC digital output accessory sends advance pulses to the detector after a specified number of motor steps.

We hope this report will be useful to the broader synchrotron radiation community and beyond.
